# Chinese college freshmen’s mental health problems and their subsequent help-seeking behaviors: A cohort design (2005-2011)

**DOI:** 10.1371/journal.pone.0185531

**Published:** 2017-10-17

**Authors:** Fenge Liu, Nan Zhou, Hongjian Cao, Xiaoyi Fang, Linyuan Deng, Wenrui Chen, Xiuyun Lin, Lu Liu, Huichun Zhao

**Affiliations:** 1 Institute of Developmental Psychology, Beijing Normal University, Beijing, China; 2 Counseling Center, Central University of Finance and Economics, Beijing, China; 3 School of Education, Guangzhou University, Guangzhou, China; 4 Psychological and Behavioral Research Center of Cantonese, Guangzhou University, Guangzhou, China; 5 New York University, New York, New York, United States of America; University of Texas Health Science Center at San Antonio Cancer Therapy and Research Center at Houston, UNITED STATES

## Abstract

Based on cohort data obtained from 13,085 college freshmen’s (2005 to 2011) SCL-90 (the Symptom Check-List-90) reports and their subsequent 4-year psychological counseling help-seeking records, this study examined the association between college students’ mental health problems and help-seeking behaviors across four college years. Female students’ mental health problems and help-seeking behaviors increased from the 2005 to the 2011 cohorts and no changes emerged for male students across cohorts. Overall, male students reported higher levels of mental health problems than did female students in the first college year, whereas female students reported more help-seeking behaviors than did male students in the following four college years. College students’ mental health problems was associated positively with help-seeking behaviors. College students were more likely to seek help from the college psychological counselling center when they experienced relatively few or quite a lot of mental health issues (i.e., an inversed U shape). Implications for future studies and practices are discussed.

## Introduction

During the past 10 years, Chinese society has undergone profound and far-reaching changes. Within the higher education domain, one of the most notable changes has been the reform of college students’ mental health education, which has expanded and improved dramatically since the initiation in Mainland China in the middle 1980s [1]. Starting from the beginning of the 21^st^ century, the Chinese government has placed great emphasis on college students’ mental health education. For example, in 2001, the Chinese Ministry of Education issued “the Opinion about Strengthening the College Students’ Mental Health Education” that firmly asserted that colleges should make efforts in providing and improving psychological guidance and consultation services for students and different departments should cooperate with each other to facilitate students’ mental health [2]. In 2005, the Chinese government further released “the Opinion about Strengthening and Improving College Students’ Ideological and Political Education” that stressed the necessity and importance of establishing sound college mental health education and consultation services, recruiting a group of part-time or full-time teachers particularly for college students’ mental health education [3]. With the support of the additional national policies, mental health education and psychological counseling services have been widely provided and promoted in Chinese universities during the following decade. However, few empirical studies have been conducted to examine current college students’ wellbeing and their counseling-seeking behaviors under this background. As such, this study attempts to examine college students’ mental health problems, help-seeking behaviors, and their associations across cohorts since 2005 to better understand, evaluate, and implement mental health education and psychological counseling services for Chinese college students.

### Mental health problems among college students across cohorts

Mental health problems are quite common among college students [4–6]. In 2008, the National Health Evaluation initiated by American College Health Association-National College Health Assessment (ACHA-NCHA) demonstrated that more than 1/3 of college students were quite frustrated and approximately 1/10 of the students considered suicide at least once during the past year due to the severity of their problems [7]. Moreover, an investigation of 26,000 students from 70 U.S. colleges found that 6% of undergraduates and 4% of postgraduates had seriously considered suicide in the past 12 months [8]. Despite the variety of samples and tools in evaluating the severity of mental health problems, Chinese college students also show a high incidence of mental problems [9–11]. A survey of 126,000 Chinese colleges students found that 16%-30% of these students suffered from depression, anxiety, obsessive-compulsive disorder, interpersonal relationship problems, personality disorders, or other mental health problems [12].

Although many studies attest to the prevalence of mental health problems among college students, it is not clear whether and how college students’ mental health problems may vary over these years or across different age groups. A report by the ACHA-NCHA stated that the proportion of U.S. college students diagnosed with the depressive disorder has increased by 10%-15% since 2000 [7]. In a national survey of the United States that used two samples from 1991 to 1992 and from 2001 to 2002, the percentage of severe depression among different age groups ranged from 3.33% to 7.06% [13]. Using a cross-temporal meta-analysis, Twenge analyzed changes in the characteristics of American college students’ anxiety between 1952 and 1993. She found that American college students’ anxiety scores increased by one standard deviation in that time period [14].

After a period of suspension during the Cultural Revolution, Chinese researchers started the research on college students’ mental health problems in the 1980s. Since the middle 1990s, Chinese researchers have begun paying attention to the cohort differences of college students’ mental health problems [15–17]. In many of these studies, the SCL-90 is widely applied as a measurement tool. These studies can be classified into three categories. First, some studies compared mental health problems of a certain grade across several cohorts. This includes early studies suggesting that the SCL-90 scores of the freshmen of 1995 and 1996 were lower than those of 1994 [18]. When looking at undergraduates as a whole, however, some studies have found conflicting results. For example, Sun and Wei sampled graduating college students from a key college in Beijing and found a yearly increase in the number of graduates with mental health problems from the 2007 cohort to the 2009 cohort (21.4%, 23.6%, and 29.23% for each cohort) [15]. Second, some studies employed longitudinal designs to capture college students’ developmental changes of mental health over time. For instance, Wang et al. conducted a multi-year longitudinal study of the class of 1996 at The Fourth Medical University, and found significant increases in students’ SCL-90 scores over time [19]. Xiao and Meng, however, found significant decreases in the SCL-90 scores of junior college students at Anhui Medical University one year later [20]. Third, some meta-analytic studies synthesized studies concerning college students’ mental health across a relative longer span of time. For example, Xin and his colleagues collected research on Chinese college students’ mental health between 1986 and 2010 and conducted a cross-temporal meta-analysis [17]. They found that Chinese college students’ SCL-90 scores generally decreased over the 25 years although they fluctuated a bit over time. This finding contradicts anecdotal evidence from many counselors and psychologists working in college counseling centers that there seems to be an upward trend in the number of students with mental health problems.

Overall, these studies suggested a lack of consensus in terms of the changes in Chinese college students’ mental health problems across cohorts. Several reasons may account for this disparity. First, these cross-sectional, historical studies may differ by sample sizes and the composition of the samples [21–23]. Most of the studies utilized samples from colleges in some specific regions. There may be a “regional integration effect” as college students’ mental health may differ across regions. Second, prior studies generally have utilized cross-sectional or short-term longitudinal designs (e.g. over 2 or 3 years) and thus may not uncover cohort differences in college students’ mental health problems. To fill this gap in the literature, this study used data obtained from 13,085 Chinese college freshmen’s (2005 to 2011) SCL-90 (the Symptom Check-List-90) reports to examine the potential differences in freshmen students’ mental health problems across cohorts.

### College students’ help-seeking behaviors

College students generally have two ways to seek help under circumstances of mental health issues: 1) The non-professional approach, including asking for help from family members, friends, and so on; 2) The professional approach, that is, asking for helps from mental health and counseling institutions. Although mental health problems are very common among college students, domestic and foreign studies revealed that only a low proportion of students used the professional approach [7,24]. For instance, the ACHA-NCHA, initiated by American College Health Association, found that only 24% of college students with depression attended therapies [7]. Comparing with foreign countries, Mainland China has paid relatively less attention to college students’ mental health issues and the provision of counseling service for them. Chinese generally show few help-seeking behaviors in the face of mental health problems. People with mental health problems account for 63% of suicide deaths in China, with only 7% having sought professional helps before committing suicides [24]. Similarly, when college students encountered mental health problems, they tended to solve them on their own. Professional helps are the last option on their mind [25–28]. Females are more likely to seek professional helps than males [25]. Females also hold more positive help-seeking attitudes than males and they are more likely to recognize and accept psychological counseling services [29–31].

Few studies outside China are available concerned with cohort differences of college students’ help-seeking behaviors. Only one has used a meta-analytic method to examine changes in American college students’ help-seeking attitudes over a 40-year period across cohorts (1968–2008) [32]. This study found a decreasing trend in American college students’ help-seeking attitudes as they adopted increasingly negative attitudes towards mental health help-seeking [32]. These results are inconsistent with those from a broad epidemiological survey on adults’ help-seeking behaviors. The Epidemiologic Catchment Area Study of help-seeking behaviors found that in 1985, only 19% of interviewees with the mental health problems received psychotherapy within the past year. In 1992, the data collected by the National Comorbidity Survey found that only 25% of interviewees with mental health problems attended psychotherapy within the past year (Kessler, Zhao, Katz, et al., 1999) [33], suggesting an increase in help-seeking behaviors. A survey conducted in 2002 from the National Comorbidity Survey Replication indicated a continuous increase in help-seeking behaviors in America between the 1990s and 2000s [34]. In addition, many studies of outpatients have found increases in help-seeking behaviors across cohorts. For instance, an American medical expenditure survey found that 0.73% patients with depression attended therapies in 1987; this number increased to 2.33% in 1997 [35], 2.37% in 1998, and 2.88% in 2007 [36].

No studies, to our knowledge, have systematically examined the cohort differences of college students’ help-seeking behaviors in Mainland China. Most studies that examined help-seeking attitudes and intentions failed to address changes across cohorts. Research showed that a relatively low rate of undergraduates in Beijing and Shanghai has ever sought professional psychological counseling such that only 7.8% students saw psychiatrists when they experienced serious insomnia or other mental problems [37]. A more recent study collected data on college students’ mental health and help-seeking behaviors from Jiangxi every 5 years between 2001 and 2011 [38]. This study found an increase in the proportion of boys’ help-seeking intentions, rising from 34.1% in 2001 to 48.2% in 2005 and reaching 62.3% in 2011. The proportion of the girls’ help-seeking intentions increased from 46.3% in 2001 to 59.4% in 2005, and reached 73.3% in 2011 [38]. Thus, in the past decade, college students in Mainland China showed an increasing trend in the acceptance and the actual behaviors of seeking psychological counseling. However, it is still not clear how exactly these professional help-seeking behaviors have changed and developed across cohorts.

Clearly, there is a lack of empirical studies focusing specifically on the cohort differences of college students’ mental health and help-seeking behaviors. This study aims to examine the changes across cohorts in college students’ help-seeking behaviors during a period of rapid development in mental health education in China. This will provide vital data to help evaluate the current mental health education by examining how mental health status relates to help-seeking behaviors among college students.

Many studies have shown that people hold some stigma toward psychological counseling, believing, for example, that people who seek for psychological counseling will not be welcomed or accepted by peers and the society [39–40]. Such beliefs can bias students’ self-reports of mental health help-seeking behaviors as students may fail to report their visits to counseling centers due to concerns about stigma, and social desirability [41]. For this reason, help-seeking behavioral records kept by the psychological counseling center could be more objective and accurate. As such, this study used actual records of help-seeking behaviors from the psychological counseling center rather than students’ self-reports.

### Mental health problems and help-seeking behaviors

Researchers have long debated about the association between the mental health problems and help-seeking behaviors. Either Cramer’s [42] help-seeking model or Gurin, Verof and Feld’s [43] three-phase process model both suggest that mental health problems are important inducing factors for help-seeking behaviors [42–43]. In general, the negative consequence of mental health problems for individuals’ lives increases when mental health problems sustain or become more serious. Thus, their mental health problems may motivate them to seek professional helps [44–45]. However, some other studies have claimed that increases in the severity and duration of mental health problems can weaken help-seeking intentions and behaviors [46–48]. Some Chinese scholars have proposed a curved U-shape to represent the relationship between mental health problems and help-seeking behaviors. Specifically, when problems are not obvious or extremely severe, help-seeking intentions and behavior are relatively low; when problems are moderately severe, help-seeking behaviors are also high; when problems are extremely severe, help-seeking behaviors may decrease as sufferers give up [49].

These studies mostly used a cross-sectional design to examine the association between the mental health problems and help-seeking behaviors. Thus, these studies actually examined the relationship between mental health problems and *prior* help-seeking behaviors and thus were not able to examine how mental health problems may predict subsequent help-seeking behaviors. To address this gap, this study examines the relationship between college students’ mental health problems in the freshmen year and their help-seeking behaviors in the following 4 years of college.

### College students’ mental health problems and help-seeking behaviors in China

Particularly, it is important to examine Chinese college students’ mental health problems and help-seeking behaviors due to the specific Chinese culture contexts. Psychological counseling was not widely available or accepted by the public in Mainland China until very recently [50]. Traditional Chinese beliefs about mental illness included the idea that mental health problems are the product of a previous sin or retribution for an ancestor’s offenses to the greater cosmic order [51]. This has produced significant mental health stigmas that may discourage help-seeking behaviors. Social and economic reform policies beginning in the late 1970s wrought considerable cultural changes. As higher education institutions also underwent significant reforms, mental health education has become a pressing responsibility. Mental health education in China has marched from adapting a foreign-origin system toward establishing a culture-sensitive educational system. For example, unlike many foreign colleges, Chinese colleges are organized in the forms of classes and homerooms. Due to the pressures of primary and secondary school entrance examinations, many Chinese freshmen are quite immature and may be at great risk for the development of mental health problems during the transition to college. In light of this, Chinese colleges place special emphases on early-phase screening and prevention education. Although these screening procedures have been implemented for over 20 years, few studies have systematically analyzed the resulting survey data. Thus, it is not clear about college students’ current mental health status. In addition to surveys, colleges also implemented other approaches, including mental health courses and lectures to popularize knowledge about mental health and to encourage students to actively seek help in the face of mental health problems. A survey of 38 Beijing colleges by the 2012 Psychological Counseling Meeting of the Beijing Higher Education Association, for example, found that all the colleges have psychological counseling centers, where services are free (students are only charged if they seek help outside the school). About 78.95% of the colleges have compulsory mental health courses and all the colleges provide elective courses. An overwhelming 93% of colleges have carried out surveys on freshmen’s mental health. Under this background, whether and how college students’ mental health problems and help-seeking behaviors vary across cohorts deserves detailed examinations.

### The present study

This study seeks to examine mental health problems and help-seeking behaviors particularly among college freshmen because of the following considerations. Freshmen are in a transitional period characterized by more freedom, academic challenges, and self-responsibility [10]. As such, upon entering college, freshmen may face many difficulties in their interpersonal relationships, academic pressures, self-management, and campus life [52]. While trying to adapt to their new college life, a number of Chinese freshmen develop some mental health problems [53–54]. By focusing on these freshmen, this study is socially necessary for developing better mental health education and services in Chinese colleges.

## Materials and methods

### Participants

This study used a multi-year cohort design and data were based on 1) a mental health survey given to college freshmen every year between 2005 and 2011, and 2) records of freshmen’s subsequent psychological help-seeking behaviors in their next 4 years of college. Both data sets were collected by a psychological counseling center in a university, Beijing. A total of 13,085 freshmen (4,985 boys with an average age of 18.41, and 8,100 girls with an average age of 18.32) participated in this survey. Table 1 shows the number of participants.

**Table 1:** The number of participants across cohorts and genders.

	Year							
	2005	2006	2007	2008	2009	2010	2011	Total
Female	830	922	1035	1073	1413	1287	1540	8100
Male	513	528	618	698	853	846	929	4985
Total	1343	1450	1653	1771	2266	2133	2469	13085

### Measures

#### Mental health problems

College students’ mental health problems were assessed using the Symptom Check-List-90 (SCL-90 for short). The SCL-90 is a 90-item self-report symptom inventory designed to screening a broad range of psychological problems. Each of the 90 items is rated on a five-point Likert scale from 1 = not at all to 5 = extremely. The SCL-90 contains 10 primary factors/subscales: Somatization, obsessive-compulsive, interpersonal sensitivity, depression, anxiety, hostility, phobic anxiety, paranoid ideation, psychoticism, and a category of “additional items” which helps clinicians assess other symptoms (e.g. poor appetite). Items were summed, with higher total scores indicating more serious mental health problems. Generally speaking, the scoring index includes total scores (90 items added together), the number of positive items (with symptoms), and factor mean scores (factor marks ≥ 2 or ≥ 3) [55]. In this study, factor marks ≥ 3 is considered a positive factor. The internal consistencies of this scale ranged from 0.88 to 0.97.

#### Help-seeking behaviors

Using psychological counseling records from a counseling center, if a student ever sought psychological counseling druing their four years at college, her/his help-seeking behavior would be recoded as 1; if there was no record of counseling, her/his help-seeking behavior would be recoded as 0.

#### Basic demographic variables

College students’ demographic information was collected, including gender, age, origin of birth, and ethnicity.

### Procedures

The counseling center at this college gives a survey screening for mental health problems to freshmen students in November each year (i.e. two months after freshmen start college), and takes records of students’ help-seeking behaviors throughout their four years’ college. This study focused freshmen surveyed from 2005 to 2011. All participants signed informed consent forms before the survey and each received a token of thanks at its completion. These gifts are pens, warm water bags, or lamps. All participants chose one of them according to their will. The survey was filled out on a computer and lasted approximately 40 minutes. The college counseling center staff explained to all participants about the aim of the study.

### Ethics statement

The study was approved by the ethical review committees of Beijing Normal University. Written consent forms were obtained from all the participants.

### Data analysis

Chi-square tests and Multivariate Analyses of Variance (MANOVA) were used to examine the gender and cohort differences of college students’ mental health problems and help-seeking behaviors. Chi-square tests and logistic regression analyses were used to examine the association between college students’ mental health problems and help-seeking behaviors. All statistical analyses were conducted using the Statistical Package for the Social Science (SPSS), version 19.0.

## Results

### Changes across cohorts and gender differences of college students' mental health problems and help-seeking behaviors

**Fig 1:**
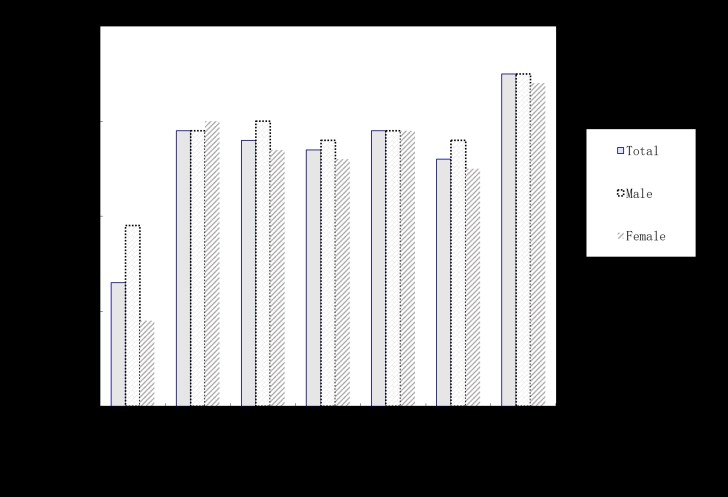
Total mean scores of SCL-90 of freshmen from 2005 to 2011.

The descriptive statistics of the major variables were displayed in Fig 1 and Table 2. The cohort and gender differences of mental health problems were examined. The total scores of SCL-90 varied as a function of either time [F_(6,13083)_ = 45.55, *p* < 0.001, η^2^ = 0.019] and gender [F_(1,13083)_ = 13.99, *p* < 0.001, η^2^ = 0.001]. Multiple comparisons showed that the 2005 cohort freshmen’s mental health problems were significantly fewer than other cohorts’; the 2011 cohort students’ mental health problems were higher than other cohorts’. Significant gender differences emerged in the 2005 and 2010 cohorts, with boys showing more mental health symptoms than girls. There were no significant differences in other cohorts.

**Table 2:** Descriptive statistics of major variables.

Year	*N*	Mental health problems *M(SD)*				The number and percentages of positive symptoms (%)			The number and percentages of help-seeking behaviors (%)		
		total	male	female	*F*	total	male	female	total	male	female
total	13085	1.47 (.38)	1.49 (.40)	1.47 (.36)		817 (6.24)	369 (7.40)	448 (5.53)	666 (5.09)	180 (3.61)	486 (6.00)
2005	1343	1.33 (.32)	1.39 (.35)	1.29 (.29)	61.78***	59 (4.39)	37 (7.21)	22 (2.65)	35 (2.61)	8 (1.56)	27 (3.25)
2006	1450	1.49 (.36)	1.49 (.37)	1.5 (.35)	.46	79 (5.45)	30 (5.88)	49 (5.53)	53 (3.66)	15 (2.84)	38 (4.12)
2007	1653	1.48 (.39)	1.50 (.40)	1.47 (.38)	2.65	121 (7.32)	52 (8.41)	69 (6.67)	55 (3.33)	19 (3.07)	36 (3.48)
2008	1771	1.47 (.37)	1.48 (.38)	1.46 (.37)	1.12	116 (6.55)	56 (8.02)	60 (5.59)	100 (5.65)	28 (4.01)	72 (6.71)
2009	2266	1.49 (.35)	1.49 (.38)	1.49 (.33)	.00	115 (5.08)	52 (6.10)	63 (4.46)	132 (5.83)	38 (4.45)	94 (6.65)
2010	2133	1.46 (.37)	1.48 (.42)	1.45 (.34)	3.94*	135 (6.33)	68 (8.25)	67 (5.34)	141 (6.61)	34 (4.02)	107 (8.31)
2011	2469	1.55 (.41)	1.55 (.42)	1.54 (.40)	2.96	192 (7.78)	74 (7.97)	118 (7.66)	150 (6.08)	38 (4.09)	112 (7.27)
*F/χ*^*2*^			13.25***	42.62***			6.67	32.49***		10.70	47.21***
		45.55***	13.99***		2.64**	28.13***	18.43***		52.81***	36.46***	

Note.

* *p* < .05,

** *p* < .0l,

*** *p* < .001.

According to the screening standards of SCL-90, factor mean score ranges from 1 to 5. If a given factor mean score is ≥ 3, this factor is considered as a positive factor [55]. On average, 6.24% of freshmen on average had at least one positive during 2005 to 2011. Among them, the lowest detection percentage was 4.39% in 2005 and the highest was 7.78% in 2011. Chi-square tests showed the detection percentage of freshmen’s positive factor differed across cohorts (*χ*^*2*^ = 28.13, *p* < .001). The average detection percentage of female students’ positive factor from 2005 to 2011 was between 2.65% and 7.66%, and male students between 5.88% and 8.41%. Chi-square tests indicated that males’ detection percentage was significantly higher than females’ (*χ*^*2*^ = 18.43, *p* < .00l). From 2005 to 2011, the percentage of female students’ mental health problems was 5.53% on average, with significant differences across cohorts (*χ*^*2*^ = 32.49, *p* < .001); the positive percentage of male students’ was 7.40% on average, with no significant differences across cohorts (*χ*^*2*^ = 6.67, *p* = .34). This suggests that in the past seven years, there were no obvious changes in male students’ positive factor whereas female students’ positive factor increased across cohorts (Table 2).

As showed in Table 2, from 2005 to 2011, the percentages of participants with help-seeking behaviors was between 2.61% and 6.61%. Chi-square tests showed significant differences across cohorts (*χ*^*2*^ = 52.81, *p* < .001). The percentages of female students’ seeking help was between 3.25% and 8.31%, and male students between 1.56% and 4.45%. On average, the percentage of females’ with help-seeking behaviors was significantly higher than males’ (*χ*^*2*^ = 36.46, *p* < .00l). The percentage of females with help-seeking behaviors also significantly differed across cohorts (*χ*^*2*^ = 47.21, *p* < .00l) whereas males’ did not (*χ*^*2*^ = 10.70, *p* = .13).

### The positive versus negative group membership and the percentage of help-seekers

According to SCL-90 test results, participants were divided into a positive factor group (any factor score ≥ 3) and a negative factor group (any factor score < 3). Table 3 shows the relationship between the positive versus negative factor group membership and the percentages of help-seekers. In the positive factor group, 14.44% of people (118 out of 817) has sought professional helps, which is higher than the 4.47% (i.e., 544 out of 12,268) in the negative factor group (*χ*^*2*^ = 6.11, *p* < .001). Although the percentage of help-seekers in the positive factor group was significantly higher than that in the negative factor group, the percentage was still quite low. However, it is important to note that some freshmen without significant mental health issues (i.e., the negative factor group) had used counseling services as well.

**Table 3:** The relationship between the positive and negative factor groups and the number of help-seekers.

Year	The positive factor group			The negative factor group		
	Total	Number and percentage of participants seeking help	Number and percentage of participants not seeking help	Total	Number and percentage of participants seeking help	Number and percentage of participants not seeking help
2005	59	3 (5.08)	56 (94.92)	1284	32 (2.49)	1252 (97.51)
2006	79	10 (12.66)	69 (87.34)	1371	43 (3.14)	1328 (96.86)
2007	121	10 (8.26)	111 (91.74)	1532	45 (2.94)	1487 (97.06)
2008	116	15 (12.93)	101 (94.86)	1655	85 (5.14)	1570 (94.86)
2009	115	21 (18.26)	94 (81.74)	2151	111 (5.16)	2040 (94.84)
2010	135	22 (16.30)	113 (83.70)	1998	119 (5.96)	1879 (94.04)
2011	192	37 (19.27)	155 (80.73)	2277	113 (4.96)	2164 (95.04)
Total	817	118 (14.44)	699 (85.56)	12268	548 (4.47)	11720 (95.53)
*χ*^*2*^	6.11***					

Note.

*** *p* < .001.

### The number of positive factors and help-seeking behaviors

**Fig 2:**
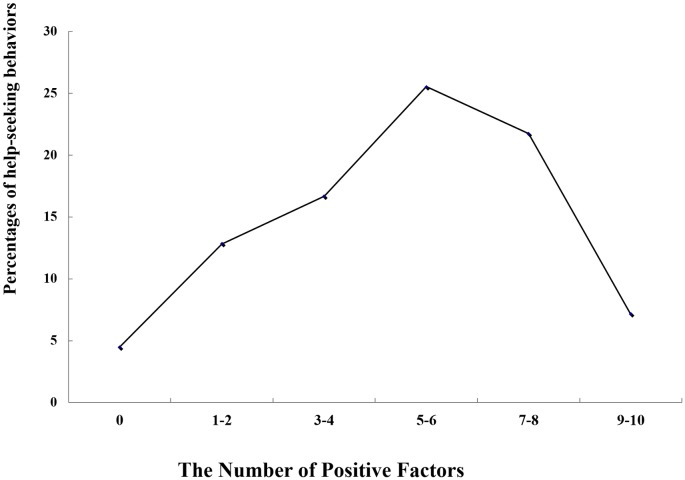
The relationship between the number of positive factors and help seeking behaviors.

The number of the positive factors were summed up to obtain the number of positive problems (ranged from 0 to 10). Given that few participants had sought help when using a single positive factor, we combined into groups of participants with the factor positive number 1 and 2, 3 and 4, 5 and 6, 7 and 8, and 9 and 10, respectively, and calculated the number and percentages of participants with and without help-seeking behaviors in these groups. Chi-square tests on the proportion of help-seeking behaviors in the number of positive factors, 1 and 2, 3 and 4, 5 and 6, 7 and 8, 9 and 10 found no significant differences between either pair (see Table 4 for specific chi-square values and *p* values). When the number of the positive factors reached 5 or 6, help-seeking behaviors increased as a whole; when the number of positive factors was larger than 5 or 6, help-seeking behaviors decreased, resulting in an inversed “V-shape” association between the number of the positive factors and help-seeking behaviors (Fig 2). In the groups of college students with differential numbers of positive factors, there were significant differences in the proportion of overall help-seeking behaviors (*χ*^*2*^ = 178.58, *p* < .001). This indicated that the number of the positive factors significantly related to help-seeking behaviors. There were also significant differences across genders in the proportion of help-seeking behaviors in the groups with differential numbers of positive factors (*χ*^*2*^ = 79.61, *p* < .00l, for males; *χ*^*2*^ = 134.26, *p* < .00l, for females).

**Table 4:** The relationship between the number of positive factors and the number of help-seekers.

The number of positive factors	*n*	The number and percentages of participants seeking help	Boy			Girl			*χ*^*2*^	*p*
			*n* (%)	The number and percentages of participants seeking help	The number and percentages of participants not seeking help	*n* (%)	The number and percentages of participants seeking help	The number and percentages of participants not seeking help		
0	12268	548 (4.47)	4616 (37.63)	139 (3.01)	4477 (96.99)	7652 (62.37)	409 (5.35)	7243 (94.65)		
1–2	577	74 (12.82)	254 (44.02)	28(11.02)	226 (88.98)	323 (55.98)	46 (14.24)	277(85.75)	6.68	0.35
3–4	156	26 (16.67)	76 (48.72)	7 (9.21)	69 (90.79)	80 (51.28)	19 (23.75)	61 (76.25)	2.59	0.13
5–6	47	12 (25.53)	19 (40.43)	2 (10.53)	17 (89.47)	28 (59.57)	10 (35.71)	18 (64.29)	1.58	0.27
7–8	23	5 (21.74)	14 (60.87)	4 (28.57)	10 (71.43)	9 (39.13)	1 (11.11)	8 (88.89)	1.57	1
9–10	14	1(7.14)	6(42.86)	0 (0)	6 (100)	8 (57.14)	1 (12.50)	7 (87.50)	1.94	0.36
Total	13085	666 (5.09)	4985 (38.10)	180 (3.61)	4805 (96.39)	8100 (61.90)	486 (6.00)	7614 (94.00)		
*χ*^*2*^		178.58***	79.61***			134.26***				

Note.

***p < .001.

To further examine the association between the number of positive factors and help-seeking behaviors, a logistic regression analysis was conducted. Gender, the number of positive factors, and time were specified as independent variables and help-seeking behaviors as dependent variables. As college students’ help-seeking behaviors significantly differed across genders and cohorts, gender and time were included step by step to check if the number of positive factors related to students’ help-seeking behaviors conditioning by gender and time. In Table 5, after controlling gender and time variables, the number of positive factors still was associated positively with students’ help-seeking behaviors.

**Table 5:** The number of mental health problems and college students’ help-seeking behaviors (*N* = 13085).

	Variables	*R*^2^	Δ*R*^2^	*B*	*SE*	*p*
Step 1		.02	.02			
	Gender			- .54***	.09	< .001
	Year			.14***	.02	< .001
Step 2		.04	.02			
	The number of positive factor			.31***	.03	< .001

*Note*. Gender is a dummy variable: Female = 0, Male = 1.

*** *p* < .001.

## Discussion

### A rising trend in college freshmen’s mental health problems across cohorts from 2005 to 2011

This study found an overall increase in mental health problems among college freshmen across cohorts from 2005 to 2011. This result is consistent with prior research concerning college students’ anxiety issues such that American college students’ anxiety scores increased from 1952 toward 1993 [14,21]. Similarly, Xin et al. found that Chinese college students’ anxiety increased across cohorts from 1993 to 2009 [21]. This also is consistent with a series of transverse historical studies suggesting gradual increases in negative psychological features or indices (e.g. mental problems, anxiety, and depression) across cohorts [56–58]. However, Xin et.al. [17] examined changes in college students’ mental health problems between 1986 and 2010 and found that college students’ mental health problems changed virtually among non-freshmen whereas freshmen showed nearly no changes across cohorts [17]. The conflicting findings may derive from the utilizations of differential samples and approaches (i.e., Xin used the transverse historical analysis) [22,23,59].

Several factors may account for college students’ increases in mental health problems across cohorts [23]. Since 2005, the Mainland China’s economy and higher education have experienced enormous changes, including rapid rises in housing prices, oversaturated job market, un- or under-employment of college graduates, and widening gaps between the rich and the poor. These factors may contribute to college students’ increased stress when they are in the midst of an already challenging transition period. This is particularly true as the mental health survey is administered only two months after the beginning of the school year when many freshmen have not settled down or adapted to the new college life. These mental health problems also may be inherited from high schools. However, some researchers argued that this increase may be explained by a concurrent increase in help-seeking behaviors [5]. Indeed, it is also conceivable that college students are becoming more aware of mental health problems than before and thus the reported mental health problems were higher over time across cohorts.

In addition to the general trend, in 2006, there was a sudden increase in mental health problems than 2005 that could be induced by the introduction of new majors, expanded enrollment, and diversified methods of enrollment. The teaching methods of these new majors are different from those of previous majors. All the courses are taught in English with high academic demands. These English courses and potentially challenging school-work may have posed extra academic pressure for those freshmen. Studies have found that the proportion of occurrence of abnormal state of mind among students who suffer from setbacks in their schoolwork was found to be rather high [60]. In 2006, this college also started recruiting students independently, and enrolled some students in terms of their athletic ability. This diversity of the freshmen may contribute to the increases in mental health problems that year. When freshmen’s mental health problems reached their peak in 2011, this similarly may be due to increasingly enrollment and more competitive enrollment scores. According to enrollment reports from this college, enrollment scores in 2011 were higher than the local minimum scores for applying to other similar key universities, and were also higher than the college’s admission scores from past years.

Additionally, this study found significant gender differences in students’ mental health problems such that the overall detection rate of male students’ mental health problems was remarkably higher than their female counterparts. During the 7-year period of our study, male students did not show significant changes in mental health problems whereas females’ mental health problems differed across cohorts. With respect to gender differences, there are not consistent conclusions in the literature [14,17,61–63]. For example, in the meta-analysis on American college students’ anxiety, Twenge [14] did not found significant gender differences across cohorts. Xin et. al.’s [17] meta-analysis, however, found more improvement in Chinese college males than female students’ mental health problems over a period of 25 years. These inconsistencies may be related to different sampling and gender ratios in these studies. We found gradual increases in female students’ mental health problems, indicating the possibility that negative aspects of China’s social and economic reform policies may be particularly relevant for females’ mental health.

### Increases in college students’ help-seeking behaviors across cohorts from 2005 to 2011

From 2005 to 2011, this study found that between 2.61% to 6.61% of college participants sought helps from the university’s professional counseling centers. The percentages of college student help-seekers have increased across cohorts in the past seven years. This may be due to a variety of factors, such as a concomitant increase in students’ mental health problems and improvements in college mental health education. After the Chinese government issued the Opinion about Strengthening and Improving College Students’ Ideological and Political Education in 2005, Chinese colleges have strengthened their efforts in mental health education by (a) reviewing the freshmen’s mental health survey, (b) routinely checking on students with mental health issues, (c) carrying out peer education projects, and (d) strengthening the quality and professionalization of psychological counselors. All these efforts have led to further standardization of and improvement in professional psychological counseling services. Such policies may have greatly popularized knowledge about mental health among freshmen, improved the availability of college mental health services, raised students’ trust in the college’s psychological counseling center, and strengthened students’ help-seeking consciousness, all of which can be linked to increases in students’ help-seeking behaviors. Thus, the implementation of the national policies concerning higher education may have led directly to improvement in the psychological health education in colleges, which in turn, may promote students' mental health and help-seeking behaviors. Therefore, it is necessary for the Chinese government to initiate plans about college student’s mental health at the national level and to support the development of college mental health education, especially the team construction and professional development in college counseling centers.

This study also found that college students with mental health problems had a higher percentage of help-seekers than those without mental health problems, but the absolute percentage of the positive group was relatively low. This suggests that over time, freshmen with mental health problems may heal by themselves without treatment, possibly because they have adapted to the college life. Data showed that compared to upperclassmen, freshman and sophomore years are peak periods for the development of mental health problems. This can have serious consequences, as underclassmen with mental health problems were more likely to be suspended or drop out of school [53, 62]. That these students still do not seek professional helps may speak to a lack of mental health help-seeking consciousness among college students. This leaves room for improvement of college mental health education. Although available mental health education and services have undoubtedly helped some students, clearly many more students either do not seek help or receive help from non-professional or non-college services. This is not unusual, as many studies of Chinese students have found that students tend to try to resolve their mental health problems on their own, with professional counselors serving as a last resort [25–28].

This study also found that 4.43% of college students without significant mental health issues had utilized psychological counseling services in the following four years. This percentage translates into 544 students, accounting for 81.68% of the total help-seekers. To some extent, this indicates that the SCL-90 test only reflects a student’s state-like psychological status during a particular period. Students’ mental health states may change over time. Perhaps there are some other factors related to college students’ mental health status and help-seeking behaviors, such as personality factors, early separation, attachments, and relationships. Note that a single freshmen survey may not sufficiently address a full picture of college students’ mental health as students’ mental health status can change as they adapt to college life. Therefore, colleges and universities should examine the effectiveness of freshmen mental health survey in order to establish and improve the early crisis warning system.

It also is notable that although the percentage of male students’ mental health problems was higher than that of female students’, males’ help-seeking behaviors were significantly lower than females’. Male students’ help-seeking behaviors did not significantly change over the course of seven years across cohorts whereas female students’ gradually increased. This is consistent with the prior studies suggesting that female students generally hold more active help-seeking attitudes than males, and they are more likely to seek psychological counseling services for helps [29–31]. In China, this may be related to traditional notions of male identity and masculinity. That means the necessity of paying particular attention to male students’ mental health problems and facilitating their help-seeking behaviors. One of the effective way in enhancing males’ help-seeking behaviors is to strengthen the popularization education and thus enhance male students' understanding of the psychological counseling, and help them overcome shame and fear toward seeking professional helps in the presence of mental health difficulties.

### College students’ mental health problems and help-seeking behaviors

This study found a reverse “V-shape” relationship between college students’ help-seeking behaviors and the number of the positive mental health problem factors. When the number of positive factors is between 0 and 6, the percentage of help-seekers increases along with the number of positive factors; when the number of positive factors is between 7 and 10, the percentage decreases as the number of positive factors increases. Vogel et al. [63] has proposed that public stigma against mental illness can influence people’s help-seeking attitudes, which, in turn, may affect help-seeking intentions and behaviors. As such, as individuals’ mental health problems become more serious, they are correspondingly more likely to be labeled and stigmatized as mentally ill. Facing the prospect of such stigma, mental illness sufferers may hold more negative view toward help-seeking and shy away from engaging in active help-seeking behaviors. In China, where psychological problems have been linked with distorted personality and moral defects, help-seeking may be stigmatized as well, negatively impacting people’s help-seeking attitudes and behaviors [64].

Although the total number of positive mental health factors is associated with college students’ help-seeking behaviors controlling for gender and time, the effect size was quite low (i.e., 2%). This verifies various theoretical models on help-seeking behaviors, suggesting that mental health problems as only one of multiple inducing factors influencing mental health help-seeking behaviors [42–43].

### Limitations

Although the present study addresses important gaps in the existing literature on college students’ mental health and help-seeking behaviors, the sampling may limit the generalization of the findings. Drawing data from students at one key college in Beijing, we may not generalize our results to non-key colleges or colleges in other cities. Moreover, this study limited help-seeking behaviors to those involving the college psychological counseling system. Thus, our current study cannot speak to the incidence or influence of off-campus professional helps and non-professional helps. Additionally, this study used a cohort design and thus it is not possible to investigate the development of mental health problems and help-seeking behavior over the four years of college. The mental health problems were reported by students themselves and may be biased by social desirability and thus future studies are warranted to use diagnostic methods to assess college students’ mental health problems.

### Conclusions

This study found a general increase across cohorts from 2005 toward 2011 in terms of both college students’ mental health problems and help-seeking behaviors. Female students showed yearly increases across cohorts in mental health problems, but no significant changes emerged for male students. Although male students had a significantly higher rate of positive mental health problems than female students, males’ help-seeking behaviors were significantly lower. College students’ mental health problems were related to their help-seeking behaviors.

## Supporting information

S1 File Data for college students’ mental health and help-seeking behaviors. The data set included in the Supporting Information files do not contain any information that could potentially identify the study's participants.
(SAV)
